# Exploring pre-requisites for clinical learning indicators: A scoping review

**DOI:** 10.4102/curationis.v47i1.2540

**Published:** 2024-11-29

**Authors:** Evelyn B. Chilemba, Felistas Chiundira, Chrissie Phiri, Felix Chisoni

**Affiliations:** 1 Department of Nursing Education, School of Nursing, Kamuzu University of Health Sciences, Lilongwe, Malawi; 2 Department of Child Health, School of Nursing, Kamuzu University of Health Sciences, Lilongwe, Malawi; 3 Department of Midwifery, School of Maternal, Neonatal and Reproductive Health, Kamuzu University of Health Sciences, Lilongwe, Malawi; 4 The Library, Kamuzu University of Health Sciences, Blantyre, Malawi

**Keywords:** clinical learning, clinical learning indicators, self-regulation, self-directed learning, competency-based education

## Abstract

**Background:**

Understanding how clinical learning takes place and what could stand as an indicator of clinical learning is crucial. There are existing challenges in the clinical learning environment that require clinical indicators. These serve as accountability standards in settings that have challenges of human resources and material poverty. Thus, clinical indicators are pre-requisites for self-regulation and self-directedness to promote lifelong learning. The reality that exists in today’s Malawian health education institutions and clinical settings requires that those in training receive support and guidance on how essential competencies and skills can be acquired during training.

**Objectives:**

The objective of this scoping review was to identify current literature on clinical learning indicators among health professional students.

**Method:**

The Joanna Briggs Institute’s (May 2020) standards for scoping reviews including narrative synthesis were followed in the conduct of this review. The protocol was registered in the Open Science Framework https://osf.io/yj9nr.

**Results:**

The results generated seven themes on clinical learning process and these are (1) planning for learning, (2) awareness of self-directedness in clinical learning, (3) knowledge of achievement of learning outcomes, (4) educators’ evidence of students’ clinical learning, (5) students’ perspective on clinical learning, (6) students’ knowledge of achievement in practice and (7) impact of prior knowledge on clinical learning.

**Conclusion:**

Clinical learning indicators among undergraduate health professionals are essential and clinical learning should be a planned endeavour by the students before the clinical placement settings.

**Contribution:**

This study contributed to understanding clinical learning indicators and self-regulated learning practices among healthcare students.

## Introduction

Clinical learning indicators are essential in health professional education to guide self-directedness in clinical learning among health professional students so that the students can self-regulate their learning. Self-directed learning (SDL) is defined as a method where a student first acknowledges his or her learning needs and establishes learning objectives. Through selected, implemented and identified learning resources, he or she assesses the outcomes (Visiers-Jiménez et al. 2021). Healthcare organisations anticipate that healthcare professionals will actively manage their own career growth to excel in clinical settings, hence self-directed lifelong learning is a critical ability that health professional students must establish from their undergraduate studies. By engaging in SDL, nurses stay current with the latest knowledge and practices, enhancing their effectiveness as healthcare providers and ultimately improving patient outcomes. Using SDL methodologies, students actively seek for, evaluate, comprehend and use information to achieve their learning requirements. Educators serve as facilitators for students to accomplish the learning goals. This implies that SDL is the student’s responsibility; however, it does not imply that SDL is just the student’s role (Al-Moteri 2019).

Students engage in self-regulated learning (SRL), a process characterised by their independent activation and maintenance of cognitive, affective and behavioural states aligned with achieving specific learning goals (Zimmerman & Martinez-Pons 1990). It is the planning and cyclical adaptation of one’s thoughts, feelings and behaviours for the purpose of achieving one’s own objectives (Zimmerman 2000). Self-regulated students approach their coursework with assurance, assiduity and resourcefulness. They are aware of when they are knowledgeable or skilled and when they are not. Whenever necessary, they actively seek out information and take the appropriate actions to understand it. The SRL perspective, distinct in its emphasis on student autonomy, offers significant insights for both instructional practices and the organisation of educational institutions (Zimmerman 1990). In contrast to SRL, SDL prioritises student autonomy and emphasises their role in independently selecting, planning and executing learning activities. A completely competent self-directed student, on the other hand, can take charge of the learning process from beginning to end, as opposed to a self-regulated student who may rely on the educator for support. Characterised by student-directed initiative, SDL positions educators as facilitators or consultants. In this context, students assume primary responsibility for finding solutions to predetermined learning content and tasks (Brandt 2020).

### Background

The reality that exists in today’s Malawian health education institutions and the clinical facility settings requires that those in training receive support and guidance on how essential competences and skills can be acquired. The terrain is challenged with shortage of human resources, material poverty, the high disease burden including HIV and AIDS. Other studies have commented on how some educators were observed of being more concerned with achieving curriculum targets rather than promoting students’ clinical learning outcomes through their scheduled supervisory visits to guide clinical learning at the clinical sites (Baluwa et al. 2023; Mbakaya et al. 2020). Currently, the stakeholders’ anecdotal reports allude to have observed that most undergraduates are guided by clinical learning objectives during clinical placements, and these are interpreted differently by individual undergraduate students who also lack structured guidance. Further, the educators rarely do a detailed analysis of the individual students’ ability because of large student-educator ratios and long distances at the various clinical sites that require financial institutional support which the institutions cannot afford at times. Therefore, it is necessary to have clinical learning indicators to guide students in their learning and educators as well as students in assessing their performance.

A learning indicator serves as a quantifiable metric facilitating individuals, particularly educators, in monitoring progress towards the enhancement of capabilities, providing a prompt and precise means to gauge achievements, assess alterations resulting from interventions or contribute to outcome evaluations. These indicators encapsulate diverse manifestations, spanning from student behaviours and actions to the results of their endeavours. They afford educators insight into the internal dynamics of student learning processes, serving as benchmarks utilised to evaluate students’ performance by delineating the proficiency levels attained in skills and attitudes (Zamorano-García et al. 2022). Clinical indicators are pre-requisites for self-directedness in learning to promote lifelong learning. The development of clinical learning indicators to guide learning in a clinical environment among health professional students is a necessity in Malawi because the stakeholder’s anecdotal reports and observation have voiced of low levels of clinical performance among some graduates and literature has reported on improved clients’ positive outcomes when graduates are deployed in health systems.

As stated earlier, because of the paucity of human resource, 10–12 students and sometimes even more are allocated to a single educator to supervise, contrary to the 1:5 educator student ratios prescribed by the Nurses and Midwives Council of Malawi (NMCM 2013). This, compounded with a lack of finances to travel to long distance clinical sites denies students the necessary support they require during clinical practice. Therefore, development of clinical learning indicators would enhance the use of self-regulation theory tenets for the undergraduates to be emancipated. Self-regulated learning has a role in clinical learning environments and in academic achievements as a process to help students construct their cognition, motivation and control their learning. The development of clinical learning indicators shall guide students in identifying their learning moments and relevant subject content for the acquisition of key competencies necessary to their professional roles. In fact, one of the tasks of healthcare professional educators is to ensure that their undergraduate students understand their professional outcomes for each clinical placement. This can only be attained through careful analysis of the subject content, and it is imperative that students understand the subject content for clinical optimal learning experiences. Furthermore, understanding of subject content is pre-requisite for mastering competences and skills in nursing practice. Therefore, through the conduct of this research, evidence-based clinical learning indicators may be developed in line with the research question: What are the educational pathways that facilitate the acquisition of competencies and skills among students?

Clinical learning indicators may guide and help train students who will be able to apply knowledge in their practice and for their personal development in settings that do not have adequate support. Thus, clinical learning among health professional students can be brought into reality by effectively introducing clinical learning indicators that are active and student-centred, focussing attention on the needs and aspiration of students rather than the educator. Literature abounds on clinical supervision and clinical teaching (Brandt 2020; Burgess et al. 2020a, 2020b; Haruzivishe & Macherera 2021; Jouhari, Haghani & Changiz 2015) is known on how students learn in clinical settings with minimal support.

Understanding how clinical learning takes place and what could stand as an indicator of learning among students is crucial since the clinical environment is riddled with challenges especially to meet the needs of students (Siddiqui, O’Halloran & Hamdorf 2021). Towle and Cottrell (1996) affirm that medical education literature provides guidance as to what will enable learning and help develop critical skills of lifelong learning among health professionals in practice settings. These include integration of prior knowledge with new learning. Learning in real-world settings enhances application. The elaboration of knowledge through activities such as discussion, questioning, peer teaching and critique, facilitates comprehension and retention (Schmidt 1983). The clinical learning indicators will enhance students’ understanding on building prior knowledge; thus, they will be able to use the knowledge which they already possess to understand and structure new information (Towle & Cottrell 1996) and choosing meaningful connections among subject areas that helps students build on their diverse experiences. The framework that integrates the concepts of SRL, SDL and transformative learning could guide in the development of clinical learning indicators that would empower students in resource-limited settings (Ramani & Leinster 2008; Towle & Cottrell 1996).

The clinical learning indicators seek to facilitate building the subject areas to provide students with better learning opportunities. It is anticipated that individuals entering health profession education will be required to engage in reflective practices, integrating new experiences, connecting current situations with past encounters, and restructuring their present experiences through a process of reflection. Self-directed learning empowers students to undertake these tasks autonomously. The clinical learning indicators shall align with the context of students’ learning, ensuring a resemblance between the learning situation and the real-world application. Additionally, the indicators should promote knowledge elaboration (Schmidt 1983).

Effective clinical learning must have indicators that will help students to set goals for their learning in the absence of a clinical facilitator. The students should be geared to take responsibility to identify appropriate learning resources, integrate materials from different sources, manage their time, assess learning progress and study habits. The clinical learning indicators will facilitate SDL as these will provide clear and advanced information about tasks expected to be performed by those in training. Therefore, to instil a reflective and critical approach to healthcare practice, health professional educators must create learning environments that foster self-confidence, encourage questioning, promote reflection, embrace openness and support risk-taking amid uncertainty and surprise.

In Malawi, stakeholders have expressed concerns regarding a disparity between the training received by students and their performance in clinical settings (Mbakaya et al. 2020). Consequently, educational outcomes fall short of the anticipated standards, leading to a workforce perceived as inadequately prepared to deliver healthcare services in both present and future contexts. Thus, although students go for clinical learning and complete the allocation with reports of successful completion of clinical placement, their level of performance upon graduation is sub-standard. This is unacceptable considering the finances that the families and the Malawi government invest in the students’ education. Further, the ‘explosion’ of knowledge in 21st century requires healthcare workers who can reason, be creative and solve client’s problem. There is need to put in place means of identifying the occurrence of clinical learning. Inadequate structuring of clinical learning leads to fragmented teaching schedules, concerns regarding the relevance of the curriculum and a lack of interdisciplinary connections and relationships. Clinical learning indicators, if designed according to graduates’ learning outcomes, should be able to assist in training competent and safe practitioners for practice (Hodges et al. 2019; Missen, McKenna & Beauchamp 2016). The terrain of the clinical environment has challenges in supporting effective clinical learning by students. It is envisaged that clinical learning indicators which integrate concepts of the theories of self-directedness, self-regulation and transformative learning would be empowering in settings where there are minimal educator-student interactions. Hence, the impetus of this study is to explore what makes students know that they have acquired the clinical competences that gives them the confidence to provide care to clients with safety amid clinical environment challenges.

This scoping review aims at identifying current literature on clinical learning indicators among health professional undergraduate students. An initial exploration for extant reviews pertaining to this subject matter was undertaken across prominent databases and search engines, including, but not limited to, the Joanna Briggs Institute (JBI), PubMed, CINAHL databases, and Google Scholar. No protocols for a similar review were found.

### Review question

What is the level of awareness about clinical learning indicators among health professional students?

## Research methods and design

A scoping review was undertaken in adherence to the JBI’s methodological framework for scoping reviews involving narrative synthesis (JBI 2020). The protocol for this review was registered in the Open Science Framework (https://osf.io/yj9nr). Scoping reviews represent a form of evidence synthesis designed to systematically delineate and chart the extent of available evidence concerning a specific topic, field, concept or issue, frequently disregarding the source (e.g. primary research, reviews, non-empirical evidence) within or across specific contexts (Munn et al. 2022). The steps that were followed included searching strategy, study selection, data extraction, data analysis and presentation.

### Search strategy and inclusion criteria

Prior to the final search, a preliminary search (Peters et al. 2020) was conducted in PubMed, Google Scholar, CINAHL and other sources for relevant articles but also to check if there are existing scoping reviews on the same topic. An analysis of the studies that were downloaded was conducted. The authors mainly focussed on the titles, abstracts and key index terms used across the articles to come up with terms for the final search strategy. The terms were agreed upon by the first author, second author and the librarian.

An experienced librarian (F. Chisoni) conducted a search of literature across five electronic bibliographic databases (Embase, PubMed, CINAHL, Web of Science and Scopus, and other sources such as Google Scholar and ProQuest theses and dissertations databases, and Ebsco open dissertations and various pre-prints repositories). The aim was to find both published and unpublished literature according to the set criteria. The search was conducted from 04 April 2023 to 21 April 2023. Keywords and Medical Subject Headings (MeSH) terms were used to identify relevant literature. Appendix 1 depicts the key terms relevant to clinical learning indicators and SRL and medical and nursing students that were used. Furthermore, the reference lists of all incorporated studies were examined to identify any additional pertinent research. Only studies published in English were included in this review, as the researchers were not fluent in other languages and lacked the financial resources to translate them.

### Study selection and inclusion criteria

This review used the inclusion criteria using the population, concept and context (PCC) framework (Table 1) published by the JBI (Salman et al. 2022). The review included studies from 2012 to 2022. This is because after conducting a preliminary search, large volumes of studies were found that could have required more time to screen and synthesise. Limiting the time frame therefore helped to narrow down the volume of literature to be reviewed, which made the process more manageable and focussed. It was also noted that recent studies were more likely to reflect current practices and understandings in the field.

**TABLE 1:** Population, concept and context framework.

PCC element	Description
Population	Nursing, medical and allied health students; health professions students.
Concept	Exploration of clinical learning indicators.
Context	Clinical learning settings such as hospitals from across the globe.

*Source*: Adapted from Peters, M.D.J., Godfrey, C., McInerney, P., Munn, Z., Tricco, A.C. & Khalil, H., 2020, ‘Scoping reviews (2020 version)’, in E. Aromataris & Z. Munn (eds.), *JBI manual for evidence synthesis*. https://doi.org/10.46658/JBIMES-20-12

PCC, population, concept and context.

All results from the electronic databases were exported into Zotero bibliographic management software. Duplicates were then removed by the librarian (F. Chisoni). Remaining articles were exported into Google Sheets (Oualline & Oualline 2018) through which title and abstract screening was conducted. To ensure reliability of the screening process (Peters et al. 2020), a pilot test of the screening process was conducted on six articles by the four reviewers. This was critical as it helped to clarify the inclusion and exclusion criteria specified in the protocol. A title and abstract screening was later conducted by the two authors. A third reviewer was consulted to resolve disagreements (F. Chiundira). The articles’ full texts were read by the first two reviewers again against the set inclusion and exclusion criteria. In accordance with the JBI guidance for scoping reviews, critical appraisal for all included studies was not conducted (Peters et al. 2020). Decisions for inclusion and exclusion were reported by following the Preferred Reporting Items for Systematic Reviews and Meta-Analyses (PRISMA) checklist (Page et al. 2021). In order to ensure that the review strictly adheres to the JBI reporting methodology, the review followed the PRISMA extension for scoping reviews checklist (PRISMAScR) (Tricco et al. 2018).

### Data extraction

Data were extracted to address the study’s objectives and the research question (Peters et al. 2020). A charting table (Table 2) was developed and tested by all reviewers using the six selected relevant articles. This table was refined several times to ensure that it exactly addressed the study’s objectives and key questions. The key issues of interest picked from each article included: evidence for planning for learning, awareness of SDL, educator’s evidence of learning, students’ knowledge of the achievement of learning outcomes, students’ perspective on SDL and impact of prior knowledge on learning. Three reviewers were involved in the data extraction process. Where disagreements occurred because of misunderstandings, the third reviewer was allowed to resolve differences. The principal investigator (E.B.C.) conducted a random check of the extracted data to ensure accuracy.

**TABLE 2:** General characteristics of primary research publications.

S/No.	Authors and study year	Title of study	Study aim	Country and setting	Study population and sample size
1	Iyama and Maeda (2018)	Development of the SRL scale in clinical nursing practice for nursing students: Consideration of its reliability and validity	To develop the SRLSCNP for the assessment of nursing students and to validate it.	Japan	Nursing universities, national, public and private universities, 766 students
2	Markowski, Yearley and Bower (2022)	Collaborative Learning in Practice (CLiP) in a London maternity ward –A qualitative pilot study	To explore the experience of student midwives who participated in the CLiP pilot and how it compared to other placement experiences.	England, a London based hospital maternity ante- and postnatal ward	Seven midwifery students (year 1 to 3) and six trained midwifery staff (total: 13)
3	Liu and Sullivan (2021)	A story half told: A qualitative study of medical students’ self-directed learning in the clinical setting	To explicate student experiences of SDL in their clinical training and to identify the roles that local social and cultural contexts play in shaping their experiences of SDL.	USA, Harvard Medical School	15 medical students who had finished their core clerkships
4	Clouder and Adefila (2017)	Empowerment of physiotherapy students on placement: The interplay between autonomy, risk, and responsibility	Explore clinical educators’ perspectives on the importance of giving student physiotherapists increasing levels of responsibility on clinical placement, and the factors considered when giving or withholding responsibility.	United Kingdom, Faculty of Health and Life Sciences at Coventry University	26 clinical educators of physiotherapy students
5	Ziba, Yakong and Ali (2021)	Clinical learning environment of nursing and midwifery students in Ghana	Assess students’ evaluation of the clinical learning environment and the factors that influence their learning experience.	Ghana, University for Development Studies	225 undergraduate nursing and midwifery students (year 3 and 4)
6	Amod and Mkhize (2022)	Clinical support and perceived competency levels of midwifery students: A descriptive analysis	To describe the clinical support and perceived competency levels of midwifery students.	South Africa public hospitals in KwaZulu-Natal	60 midwifery students (year 4) from an undergraduate nursing programme at a University in KwaZulu-Natal
7	Rodríguez-Monforte et al. (2023)	Comparing preferred and actual clinical learning environments and perceptions of first-year nursing students in long-term care: A cross-sectional study	To assess first-year nursing students’ ‘preferred’ and ‘actual’ clinical learning environments when conducting their first placements in nursing homes.	Spain, Blanquerna School of Health Sciences in Barcelona	154 first-year nursing students in the 2022–2023 academic year
8	Olsen et al. (2014)	Evidence-based practice exposure and physiotherapy students’ behaviour during clinical placements: A survey	To compare self-reported EBP behaviour, abilities and barriers during clinical placements.	University College in Norway	180 third-year physiotherapy students
9	Kurt and Eskimez (2022)	Examining SRL of nursing students in clinical practice: A descriptive and cross-sectional study	To examine SRL towards clinical practice and the influencing factors in nursing students.	Çukurova University Faculty of Health Sciences Nursing Department, Adana, Turkey	614 nursing students
10	Al-Moteri (2019)	Self-directed and lifelong learning: A framework for improving nursing students’ learning skills in the clinical context	To identify if the current clinical placement at the Nursing Department of a Saudi University helps students to be self-directed lifelong students.	Taif University, Taif, Saudi Arabia	76 students
11	Hess, Miles and Bowker (2022)	Placement overlap with other students: Effects on medical student learning experience, teaching and learning in medicine	Investigate the impact of student-student encounters on the learning experience during clinical placements before student numbers increase further.	United Kingdom	844 medical students

Note: Please see the full reference list of this article for details on the articles cited: Chilemba, E.B., Chiundira, F., Phiri, C. & Chisoni, F., 2024, ‘Exploring pre-requisites for clinical learning indicators: A scoping review’, *Curationis* 47(1), a2540. https://doi.org/10.4102/curationis.v47i1.2540.

EBP, evidence-based practice; SDL, self-directed learning; S/No, serial number; SRL, self-regulated learning; SRLSCNP, SRL Scale in Clinical Nursing Practice.

### Data analysis and presentation

The data extraction tool provided a source of findings that informed this study. Following a recommendation for scoping reviews by JBI (Peters et al. 2020), a descriptive summary is presented with various visual representations such as tables and figures.

### Ethical considerations

This review is part of the study on Developing Clinical Learning Indicator currently being conducted in Malawi. Ethical approval to conduct this study was obtained from the University of Malawi the College of Medicine Research and Ethics Committee (COMREC) (reference no. P.05/23/4106).

## Results and discussion

### Study inclusion

**FIGURE 1:**
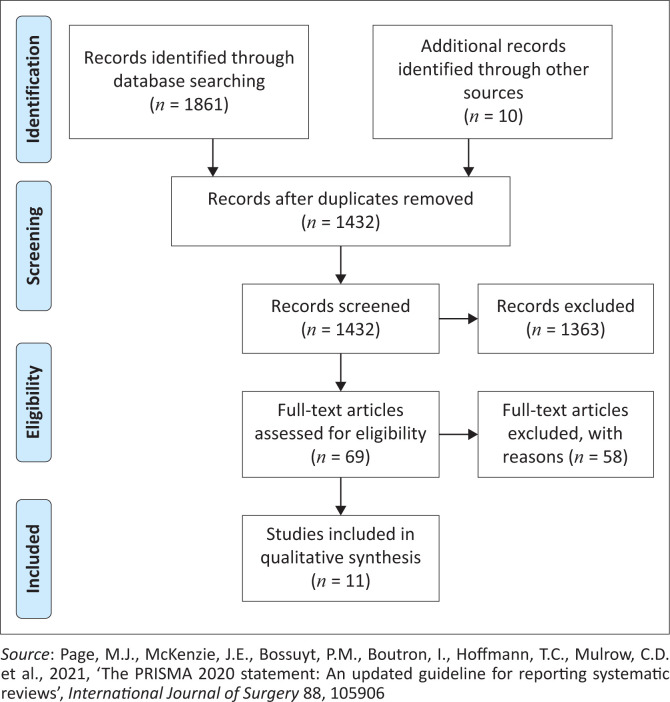
Study selection process.

The database searches identified 1861 results and after duplicates removal, 1432 remained for title and abstract screening. Ten studies were identified through checking of the reference lists and other sources. Two independent reviewers (E.B.C., C.P.) reviewed the 1432 titles and abstracts against the inclusion and exclusion criteria. After the titles and abstract screening, full texts of the 69 studies were reviewed by E.B.C. and C.P. again, resulting in the inclusion of 11 articles in this review (Figure 1).

### Characteristics of the included studies

The characteristics of the included studies (author, title of study, aim and design, country and setting, study population) are presented in Table 2.

The description of these results is provided to reflect the findings of the scoping evaluation of the 11 selected articles to answer the research questions that guided the review. Based on the initial analysis, the researchers mapped the articles directly or indirectly relevant to exploring clinical learning indicators from the perspectives of undergraduate health professionals and nurse educators. Articles that did not fall into the six categories of clinical learning indicator aspects were excluded, and this resulted in 11 being selected. The mapping results are presented in Table 3 from the journal aspect (name, edition, volume, and year). The research questions focussed on the following: planning for clinical learning, awareness of self-directedness in clinical learning, knowledge of achievement in clinical learning, educators’ evidence of undergraduate student achievement of clinical learning, students’ perspectives of clinical learning and knowledge achievement, impact of learning.

**TABLE 3:** Mapping of clinical learning indicators themes.

S/No.	Authors and study year	Planning for learning	Awareness of self-directed learning	Students’ knowledge of the achievement of learning outcomes	Students’ perspective on self- directedness	Impact of prior knowledge on learning	Students’ knowledge of achievement in practice	Educators’ evidence of students’ clinical learning
1	Iyama and Maeda (2018)	Learning strategies: Validating clinical nursing competency prior to patient care. Exploring personalised care approaches to address individual patient needs. Learning patient materials before clinical nursing practice. Enhancing nursing skills through independent practice during clinical rotations. The cognitive strategies include rehearsal (e.g. repeating words), elaboration (e.g. paraphrasing or summarising), organisation (e.g. sorting into categories), and critical thinking (e.g. critical examination, acquisition of new thoughts). Synthesis of knowledge and nursing skills. Cultivating a reflective mindset informed by clinical nursing experiences; holistic thinking: generating novel and creative solutions. Integrate prior knowledge with clinical experiences: merging recent knowledge with past experiences to develop novel perspectives; therefore, it was termed.	The respondents’ inherent motivation as evidenced in their curiosity, enthusiasm and favourable attitude towards clinical nursing practice.	Achievement motivation. The nursing student utilised a positive assessment as a stepping stone towards more ambitious objectives.		Associate what I have experienced through clinical nursing practice with the knowledge I previously acquired. Students were observed to synthesise new learning with past knowledge.	High scores guided reflections for self-evaluation.	
2	Markowski et al. (2022)	Use of CLiP model. The CLiP midwife, who is the practice supervisor, supervises three students (year 1, 2, 3) to take on care planning and delivery for a specified number of women using coaching techniques based on the GROW model: GOAL (what do you want to achieve?), REALITY (what is your current situation?), OPTIONS (what could you do?) and WAY FORWARD (what will you do?); these questions guide the coach to listen and elicit the answers from the coachee before they act.	Students perceived the CLiP hour, when managed effectively, as very beneficial for peer discussion and reflection on their practice. The CLiP hour allowed students to reinforce their learning needs as well as to inform peers and staff about their learning goals.		Students worked together to address challenges. Peer support to clarify questions, solve problems and reflect on their own practice. Students enjoyed working with fellow students; reduced anxiety levels and emotional support. Having fellow students present was perceived as a resource and allowed for collaborative problem solving.	Peer-learning and collaboration as support and re- source. The CLiP midwife facilitated students to plan, deliver and evaluate care independently.		
3	Liu and Sullivan (2021)	Identifying gaps in content knowledge and a need to learn how to apply their knowledge to specific clinical cases (real-world patient cases). Finding learning resources: online and printed resources for reading and test preparation (e.g. journal articles, visual learning YouTube videos) and Google search. Use of printed resources: textbooks, pocket manuals, and question based books.	Generating the learning topics through: Case preparation through collection of patient’s data and presenting the case on rounds or teaching sessions. Direct patient care, direct involvement in primary care and whereby students encountered a series of clinical problems. Learning from nurses, paramedics, interns, residents, physicians. Peer-learning and support through peer teaching, word of mouth, and shared resources.	Self-reflection: monitoring personal progress and accomplishments, the number of patients treated and their diagnoses. Writing down important experiences that happened during the day. Student tracks down all patients seen by him/her in terms of their conditions and diagnosis.	Participants described SDL by two dominant mechanisms: asking the team members questions and seeking their feedback.		Students were reported to have interacted on important experiences through self-reflections.	
4	Clouder and Adefila (2017)	Not relevant; one student to one educator; clinicians were used as clinical educators; hands-on teaching, demonstrating techniques, observing students’ interactions and treatments, providing feedback, questioning and testing and enhancing students’ knowledge and capabilities. Clinical educators assess the students’ mid-way, and at the end of placements in collaboration with an academic tutor. Students were expected to prepare for each placement, to be open to constructive feedback and to use their initiative to maximise their learning, by, for instance, using spare time to observe different clinicians working and asking questions when appropriate. Giving independence to students: trusting them so that they are competent. Cycle of empowerment-coaching and scaffolding.						
5	Ziba et al. (2021)			Clinical clerkship management correlated positively (*r* = 0.153) with OSCE scores, while control over learning beliefs was negatively correlated (*r* = −0.159) with OSCE scores at the statistical level of 0.1.	Learning in clinical setting, our study revealed that motivation-related personal factors and social factors related to clinical context could promote the SRL level of students. Learning motivation (extrinsic goals) and learning strategy (clinical clerkship evaluation) were positively associated with students’ clinical achievements on clinical skills. External support, such as clinical clerkship and management, might improve clinical skills in clinical setting.	The external support such as clinical clerkship management and the extrinsic goals such as grades ranking might be important for learning clinical skills in China. Students need support such as an experienced tutor to improve their clinical skills in the clinical environment.		
6	Rodríguez-Monforte et al. (2023)	Guided by the clinical mentor 30 min briefing session on objectives of the day, expectations and fears. Academic mentor accompanied the students to each of the wards where they were assigned in the nursing home, and with the clinical mentor, shared the expectations, objectives, and tasks that the students aimed to perform. At the end of the day, students had debriefing session with academic mentor.						
7	Olsen et al. (2014)			There was no statistically significant association between EBP exposure and students’ use of research evidence in clinical settings (*p* = 0.09).		Our students had just completed their final clinical placement; they would graduate 3 months later and were expected to have the competence to use research evidence in the course of patient care. They did report use of research evidence in clinical situations; 48% when EBP was not integrated and 61% when EBP was fully integrated. However, the association between EBP exposure and students’ use of research evidence in clinical settings was not statistically significant (*p* = 0.09).		
8	Kurt and Eskimez (2022)	The higher mean scores of the ‘Motivation’ sub-dimension and the ‘Success Motivation’ factor in the first-year students is an expected result. Making decisions and taking part in new experiences are the primary features of the placement learning style.	The results of this study show that SRL in clinical practice is higher in females, first-year students, students who are interested in the nursing profession, and see academicians as role models.	‘Learning Strategies Sub-Dimension’, and the ‘Success Motivation Factor’. Female students outperformed male students on the items ‘Getting good scores from clinical nursing practices is important for me’, ‘I want to get better scores from my classmates in clinical nursing practices’ and ‘I want to show my talent to others by getting good scores in clinical nursing practices’. Although the number of male students in nursing schools has recently increased, the fact that the majority of the students are female can increase competition in the learning environment.		There are studies in the literature that show an increase in SRL skills as the age of university students and their academic level increase. To increase self-regulation, students need role models that convey their experiences in clinical practice to the students in the best manner, provide information during the applications, and encourage them to ask questions.		Students were reported to have excitement and curiosity to learning.
9	Al-Moteri (2019)	The results clearly indicated that, in this university, the current clinical placement does not help nursing students to be self-directed lifelong learners.	For instance, being alone without direct supervision in clinical placement might be seen by some students as an opportunity to independently apply and practise their learned skills. By contrast, other students might see it as limiting their chance to learn because there is no instructor to provide feedback, which is one of their learning strategies in a clinical placement.				Reports on recognition in achievement helped students to cooperatively discuss clinical academic content, exchanged and shared information and knowledge. They practised skills to promote mastery of content.	Students were reported to be able to solve problems on their own on learning in practice. Learners had opportunities to learn, become passionate to master skills.
10	Hess et al. (2022)			A survey-based study of medical students’ experiences reveals that they perceive their peers in the clinical environment to significantly influence their learning, both positively and negatively. This research appears to be the first to formally document and quantify these student perceptions within a United Kingdom medical school. However, our findings likely have broader implications for any clinical setting that accommodates large groups of students.				
11	Bransen (2022)					First-year students were reported inclined to be discussing strategies for effective clinical learning with friends.	Students discussed with friends through self-reflection on important experiences.	
12	Hess et al. (2022)					Students with positive clinical experiences were more satisfied with the organisation of their clinical placement and the overall clinical experiences than those who had negative experiences. The students took advantage of shared learning time on placement to create their own ways of learning in practice.	Students large numbers on placement impacted on learning outcomes positively or negatively.	
13	Zhang et al. (2022)							Literature cite of excitement and curiosity among learners. Learners had ambition to belong to a group of learners. Educators observed increased interactions among leaners by asking questions. Learners sought answers by searching for information.

Note: Please see the full reference list of this article for details on the articles cited: Chilemba, E.B., Chiundira, F., Phiri, C. & Chisoni, F., 2024, ‘Exploring pre-requisites for clinical learning indicators: A scoping review’, *Curationis* 47(1), a2540. https://doi.org/10.4102/curationis.v47i1.2540.

CLiP, Collaborative Learning in Practice; EBP, evidence-based practice; SDL, self-directed learning; S/No, serial number; SRL, self-regulated learning.

The scoping review sought to seek evidence in self-regulation, SDL and clinical learning literature that would support the development of clinical learning indicators for undergraduate healthcare professionals. This follows the claim that students control and direct their own learning under active internal sources of self-regulation and self-directedness.

### Planning for learning

Research in clinical learning cite of planning as a critical area in promoting meaningful lifelong learning (Bransen et al. 2022; Chitra et al. 2022; Clouder & Adefila 2017; Iyama & Maeda 2018; Kurt & Eskimez 2022; Liu & Sullivan 2021; Markowski et al. 2022; Al Moteri et al. 2019; Rodríguez-Monforte et al. 2023). Bransen et al. (2022) state that students getting involved with supervisors or mentors to discuss clinical goals resulted in engagement with peers in exploring strategies and opportunities in clinical learning. Chitra et al. (2022) further highlight the significance of student proactiveness in clinical learning environments. Their research suggests that students’ capacity for forethought and goal setting is essential in shaping their learning styles. By strategically organising tasks and managing time, students create opportunities for focussed learning. This includes prioritising tasks based on urgency and identifying study areas requiring immediate attention. Notably, effective time management empowers students to achieve a healthy balance, allowing them to dedicate time for personal pursuits and relaxation. However, the value placed on peer feedback remains another key aspect of student learning.

This involved identifying errors, making corrections, giving guidance as they planned for learning which made them to reflect on their performance and to identify reasons for their success or failures in clinical learning (Chitra et al. 2022). It is also reported that planning effectively prior to placement enhanced the success of clinical learning, in terms of 30 min briefing on objectives of the day, sharing expectations, tasks and fears (Rodriquez-Monforte et al. 2020). To this end, implementing care tailor-made to individual patient’s needs was a strategy that helped students to learn materials before practice. This is a process which was regarded as cognitive rehearsal where there was elaboration, critical thinking, synthesis of nursing and skills. In support to the cognitive rehearsal process, Iyama and Maeda (2018) affirm that students develop ways of thinking based on what has been learnt thus, multidimensional thinking. A self-directed learner can determine their own learning path and strategies. Furthermore, a skilled self-directed learner identifies their learning gaps, sets learning objectives, locates relevant resources and tracks their progress (Hooshyar et al. 2022). Jouhari et al. (2015) discovered that planning mistakes, inconsistent daily scheduling and undefined goals were hindering factors for self-regulation among medical students.

Enhancing thinking is valued in health professionals because undergraduate healthcare professionals work in dynamic settings that require them to make decisions affecting patients’ lives. Markowski et al. (2022) recommend the use of a Collaborative Learning in Practice (CLiP) model in planning clinical teaching where these questions serve to guide the coach in actively listening and prompting students to articulate their own solutions before offering interventions. Hence, it is essential to identify knowledge gaps among students when planning clinical learning. Liu and Sullivan (2021) confirm the need to identify gaps in content knowledge to reinforce its application to specific clinical cases. Therefore, planning for clinical learning should include finding resources and use of printed resources. Students should be given independence in clinical learning (Clouder & Adefila 2017), educators should be open to provide constructive feedback and use space to maximise observing students’ interactions trusting their competences and the cycle of empowerment. Furthermore, learning must be planned for the learner to be motivated to succeed. Kurt and Eskimez (2022) affirm that taking part in new experiences reflects interest in the planning of learning by the educators.

### Awareness of self-directedness in clinical learning

Clinical learning literature confirms of students’ awareness of the self-directedness to advance opportunities of lifelong learning in practice (Kurt & Eskimez 2022; Liu & Sullivan 2021; Al Moteri et al. 2019; Zhang et al. 2022). Thus, students realise the opportunities existing in practice where they must learn independently to apply knowledge (Al-Moteri 2019). Chitra et al. (2022) cite of students who engaged in self-reflections after completion of each task to analyse and review their performance. According to Iyama and Maeda (2018), learner’s increased intrinsic motivation resulted in curiosity, interest, positive attitudes to clinical practice. Self-directedness empowers students to leverage their existing knowledge base for the effective comprehension and organisation of new information. This enhanced the ability to utilise prior knowledge to facilitate the construction of meaningful frameworks for new clinical concepts. Liu and Sullivan (2021) report of self-directedness of student in practice where the students had generated learning topics through case preparation from the collection of patients’ data. Markowski et al. (2022) affirm the value of the CLiP model, particularly the dedicated ‘Clip hour’. This dedicated time allows students to solidify their learning needs, share their learning goals with peers and educators, and potentially receive further guidance. According to Jouhari et al. (2015), medical students who had a foundational understanding of self-regulation were more likely to engage in SRL.

### Knowledge of achievement of learning outcome

The knowledge of achievement of clinical learning outcomes is essential for it the clinical learning experiences that students are exposed to in fulfilment of professional outcomes. A study by Bull et al. (2020) suggests that student debriefing and reflective sessions are valuable tools for promoting learning and sense-making during clinical placements. These sessions contribute to the development of critical thinking skills in students. The study also highlights the potential benefits of assigning specific daily tasks to students, as suggested by clinical facilitators. This approach is believed to foster targeted learning and enhance student achievement. Zhang et al. (2022) contend that learning motivation (extrinsic goals) and learning strategy (clinical clerkship evaluation) are positively associated with students’ clinical achievements on clinical skills. However, this study attests that external support might improve the acquisition of clinical skills. Hence, there should be a clear career plan to improve students’ self-efficacy, thereby enhancing their independent learning in the process of clinical practice. Supporting Zimmerman’s (2000) proposition that goal setting is a critical element in student self-regulation, Zhang et al. (2022) affirm this notion through their research. Doyle et al. (2017) confirm that the culture of clinical placement had enhanced the satisfaction rate among their students where the students had expressed to have felt comfort to learn. The present research identified a positive workplace culture as the most robust predictor of student-perceived success in clinical placements. This culture, characterised by strong work ethic and high team morale, significantly contributed to a successful learning experience for undergraduate student nurses.

### Educators’ evidence of students’ clinical learning

Knowledge of achievement is essential from the social cognitive perspective as an ability of an individual to manage their own behaviour in learning through observation and evaluation. Educators’ knowledge of learner’s attainment of clinical learning outcome in practice settings is evident from clinical experiences (Al-Moteri 2019; Kurt & Eskimez 2022; Zhang et al. 2022). The literature attests to the excitement and curiosity among the students to learning and that the students aspire to belong to a group of students. Furthermore, the students displayed enthusiasm in their clinical learning and the educators observed that students were asking questions and sought for answers through searching for information. This is self-regulation where the students are trying to be emancipated through important feedback. In a study which investigated the potential of the Active Clinical Training Approach (ACTA) to promote student engagement in clinical practicum settings and foster the development of SDL skills, Al-Moteri (2019) found that students believed that they were able to solve problems as they took opportunities to learn and become passionate to master prescribed skills.

### Student perspectives on clinical learning

The growing emphasis on learner-managed clinical practice necessitates a focus on SRL. To effectively support this shift, investigating students’ perspectives on their clinical learning experiences is crucial. Some studies have reported on students’ perspectives on clinical learning (Hess et al. 2022; Kurt & Eskimez 2022; Markowski et al. 2022). The studies have identified several self-regulation concepts that students themselves consider essential for success in clinical practice. These concepts include: thorough preparation through preliminary reading and planning, effective time management and organisational skills, active engagement with academic skills and resources, maintaining a positive learning attitude, proactive questioning and participation in laboratories and training sessions, strong self-discipline in approaching their learning (Kurt & Eskimez 2022). Furthermore, the student-student interactions were reported to have influence on clinical learning experiences of medical students that were both positive and negative (Hess et al. 2022). Liu and Sullivan (2021) report of students asking questions and seeking feedback on clinical learning as a sign of SDL. In support of this notion, Markowski et al. (2022) outline strategies that students took to show responsibility of their clinical learning. The students reported that they worked together to address challenges of clinical learning, used peer support for clarity of questions to solve problems and reflected on practice. This is the process that increases success in clinical practice. The development of SRL abilities and effective learning strategies are considered essential for students’ success in clinical practice. By equipping students with these skills, educators can empower them to navigate the complexities of clinical environments and ultimately achieve better learning outcomes. However, Kurt and Eskimez (2022) attest that SRL in clinical practice exists and it is higher in female students. While several studies have investigated the general SRL abilities of students, there is a scarcity of research focussed on clinical practice. Given the connection between SRL and students’ independent patient care skills and academic performance, it is crucial to promote its implementation in practical settings.

### Students’ knowledge of achievement in practice

Clinical learning is dynamic and requires that students possess some imaginations on how they can navigate the learning to attain the required professional outcomes. Students need to have knowledge on how they achieve their learning outcomes to ensure that they attain the required competences and skills. Hess et al. (2022) state of large student numbers on placements that impacted on learning outcomes, and these resulted in negative and positive impacts. Al-Moteri (2019) reports that in recognition of achievement, students cooperatively discussed, exchanged and shared information and knowledge with others. The students practised skills cooperatively to promote mastery of the content. This confirms that knowledge of achievement in clinical learning motivates students. Similarly, in their study, Clouder and Adefila (2018) report of students who used good scores for self-evaluation and tried to achieve higher scores. The learners’ interactions targeted at discussing self-reflections where they wrote down important experiences and had followed up patients (Liu & Sullivan 2021).

### Impact of prior knowledge on clinical learning

Prior knowledge has impact on clinical learning. According to Bransen (2022), first-year students were inclined to discuss learning strategies with friends. Iyama and Maeda (2018) observed students who were able to integrate their existing knowledge with new learning to create novel understanding in practice. Thus, the students associated what was experienced in clinical nursing practice with previously acquired knowledge. According to Hess et al. (2022), students who had positive clinical experiences were more satisfied with the structure of their placements and their overall clinical learning experiences than those who had negative experiences. It is essential to consider previous students’ negative experiences when designing clinical learning opportunities, as these experiences can negatively influence other students’ overall view of the placement, regardless of any positive experiences they may have had. This emphasises the need to consider the likelihood of encounters with other students’ placement planning and actively recognising areas where negative experiences may occur so as to optimise the learner clinical experience. Hess et al. (2022) observed students who seemed to have capitalised on shared time during their placements to develop their own learning opportunities in practice.

With the growing number of students in Malawian clinical settings, fostering more opportunities for informal peer clinical learning activities can be beneficial for promoting clinical learning. Therefore, it seems that earlier-year students have gained more advantages from interactions with other students compared to more senior students. Additionally, junior students perceived that students in higher years of the MBBS programme had the most positive influence on their learning.

## Conclusion

Based on the reviewed literature, it is evident that clinical learning among undergraduate health professionals should be a planned endeavour by the students before the placements in clinical settings. The students need to be aware of their responsibilities in the development of self-directedness in clinical sites to ensure that they can attain the expected outcomes. It is envisaged that the knowledge of achievement of learning outcomes could be an indication and evidence of students’ learning in clinical practice. Students’ knowledge of achievement of learning is an essential element for sound educational practices.

## Appendix 1

**TABLE 1-A1:** PubMed search strategy.

S/No.	Query	Results
4	Medical Students[MeSH Terms]) OR students, medical[MeSH Terms])) OR students, nursing[MeSH Terms])) OR nursing students[MeSH Terms] OR Allied Health Students[Text Word]O R ‘Undergraduate Students’[Text Word] OR Physiotherapy student*[Title/Abstract] OR Dental Students[Title/Abstract] OR Student, Dental[Title/Abstract] OR Midwifery students[Title/Abstract] OR pharmacy students[MeSH Terms] OR physiotherapy student[Title/Abstract] AND y_10[Filter])) AND SDL as Topic [MeSH Terms] OR SDL [Text Word] OR Clinical learning[Text Word] OR SRL [MeSH Terms] OR Clinical learning indicators [Text Word] OR ‘Clinical learning outcome’[Text Word] AND y_10[Filter]))) AND ‘student placement’[Text Word] OR ‘clinical placement’[Text Word] OR ‘student clinical placement’[Text Word] AND y_10[Filter])) Filters: in the last 10 years	130
3	‘student placement’[Text Word] OR ‘clinical placement’[Text Word] OR ‘student clinical placement’[Text Word]) AND y_10[Filter])	1196
2	Search: SDL as Topic [MeSH Terms] OR SDL [Text Word] OR Clinical learning [Text Word] OR SRL [MeSH Terms] OR Clinical learning indicators [Text Word] OR ‘Clinical learning outcome’[Text Word] Filters: in the last 10 years	6340
1	Search: Medical Students [MeSH Terms]) OR students, medical [MeSH Terms])) OR students, nursing [MeSH Terms])) OR nursing students [MeSH Terms] OR Allied Health Students [Text Word] OR ‘Undergraduate Students’[Text Word] OR Physiotherapy student*[Title/Abstract] OR Dental Students[Title/Abstract] OR Student, Dental[Title/Abstract] OR Midwifery students[Title/Abstract] OR pharmacy students[MeSH Terms] OR physiotherapy student[Title/Abstract] Filters: in the last 10 years	7925

S/No, serial number.
